# Clinical and molecular findings in a Chinese family with a de novo mitochondrial A1555G mutation

**DOI:** 10.1186/s12920-022-01276-y

**Published:** 2022-05-25

**Authors:** Ping Gu, Guojian Wang, Xue Gao, Dongyang Kang, Pu Dai, Shasha Huang

**Affiliations:** 1 College of Otolaryngology Head and Neck Surgery, Chinese PLA General Hospital, Chinese PLA Medical School, Do.28 Fuxing Road, Beijing, 100853 People’s Republic of China; 2 National Clinical Research Center for Otolaryngologic Diseases, State Key Lab of Hearing Science, Ministry of Education, Beijing, People’s Republic of China; 3 Beijing Key Lab of Hearing Impairment Prevention and Treatment, Beijing, People’s Republic of China; 4 Department of Otolaryngology, Shenzhen Children’s Hospital, Shenzhen, 518038 People’s Republic of China

**Keywords:** A1555G, De novo mutation, Mitochondrial DNA, Maternal inheritance

## Abstract

**Background:**

The mitochondrial 12S rRNA A1555G mutation is the most prevalent deafness-causing mitochondrial DNA (mtDNA) mutation and is inherited maternally. Studies have suggested that A1555G mutations have multiple origins, although there is no direct evidence of this. Here, we identified a family with a de novo A1555G mutation.

**Method:**

Based on detailed mtDNA analyses of the family members using next-generation sequencing with 1% sensitivity to mutated mtDNA, the level of heteroplasmy in terms of the A1555G mutation in blood DNA samples was quantified.

**Results:**

An individual harbored a heterogeneous A1555G mutation, at 28.68% heteroplasmy. The individual’s son was also a heterogeneous carrier, with 7.25% heteroplasmy. The individual’s brother and mother did not carry the A1555G mutation, and both had less than 1% mitochondrial 12S rRNA A1555G heteroplasmy.

**Conclusion:**

The A1555G mutation arose de novo in this family. This is the first report of a family with a de novo A1555G mutation, providing direct evidence of its multipoint origin. This is important for both diagnostic investigations and genetic counselling.

## Background

Deafness is a common health problem, affecting approximately 1 in 1000 newborns worldwide [1]. Hearing loss can be caused by genetic and environmental factors. Approximately 60% of congenital hearing impairment cases have a genetic cause, with autosomal dominant, autosomal recessive, X-linked, and mitochondrial patterns of inheritance [2]. Mutations in the *GJB2*, mitochondrial 12S rRNA, and *SLC26A4* genes play important roles in hearing loss. More than 200 point mutations in mitochondrial DNA (mtDNA) have been reported in the mtDNA mutation database MITOMAP. Since the deafness caused by A1555G mutation in the mitochondrial 12S rRNA gene was first reported by Prezant et al. in 1993, many mitochondrial 12S rRNA A1555G mutant families associated with aminoglycoside induced deafness and maternally inherited nonsyndromic deafness have been reported all over the world, with its prevalences of 2.4% in European sensorineural deafness patients and 3.2% in Chinese sensorineural deafness patients [3–5]. The A1555G mtDNA mutation in the 12S rRNA gene is the third most common deafness-causing mutation in the Chinese population [3].

The A1555G mutation is located in the aminoacyl-tRNA acceptor site of the small ribosomal subunit, which is highly conserved from bacteria to mammals [6]. Little is known about the incidence of de novo A1555G mutations. Here, we report an individual with a de novo heterogeneous A1555G mutation in 12S rRNA who presented with normal hearing. This provides direct evidence of the multipoint origin of the A1555G mutation.

## Materials and methods

### Patients

**Fig. 1:**
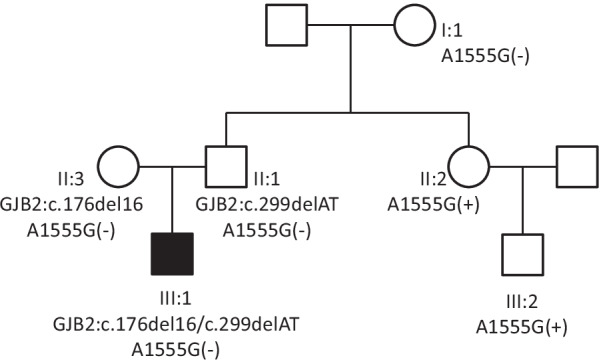
The family diagram and mutations in family 2362

We reviewed the genetic characteristics of families possessing the A1555G mutation in the database of the Genetic Testing Center for Deafness (PLA General Hospital, Beijing, China) and found a family (Family 2362) that did not fully conform to the maternal genetic characteristics (Fig. 1). As controls, 200 individuals negative for *GJB2*, *SLC26A4*, and mtDNA 12S rRNA mutations were recruited, including 100 individuals with normal hearing (Group 1) and 100 patients with sensorineural hearing loss (Group 2).

Each participant underwent a comprehensive clinical history and physical examination. Audiological examinations were performed, including pure-tone audiometry and auditory brainstem responses.

### Paternity testing

Paternity was tested for three generations (I:1, II:1, II:2, and III:2) using genotype analysis of 15 informative chromosome short tandem repeats (STRs).

### Mutation analysis

Genomic DNA was extracted from the peripheral blood of subjects using a blood DNA extraction kit according to the manufacturer’s instructions (Tiangen, Beijing, China). All family members were screened for common deafness genes, including *GJB2*, *SLC26A4*, and mtDNA 12S rRNA, using polymerase chain reaction (PCR) amplification, and the exons were sequenced directly [7]. For quantitative analysis of the mutation frequency at nucleotide 1555 in the control group and members of Family 2362, capture and next-generation sequencing (NGS) of a 147-bp DNA fragment corresponding to positions 1466–1612 of the mitochondrial 12S rRNA gene was performed on the Ion Proton System.

## Results

### Deafness gene testing

**Fig. 2:**
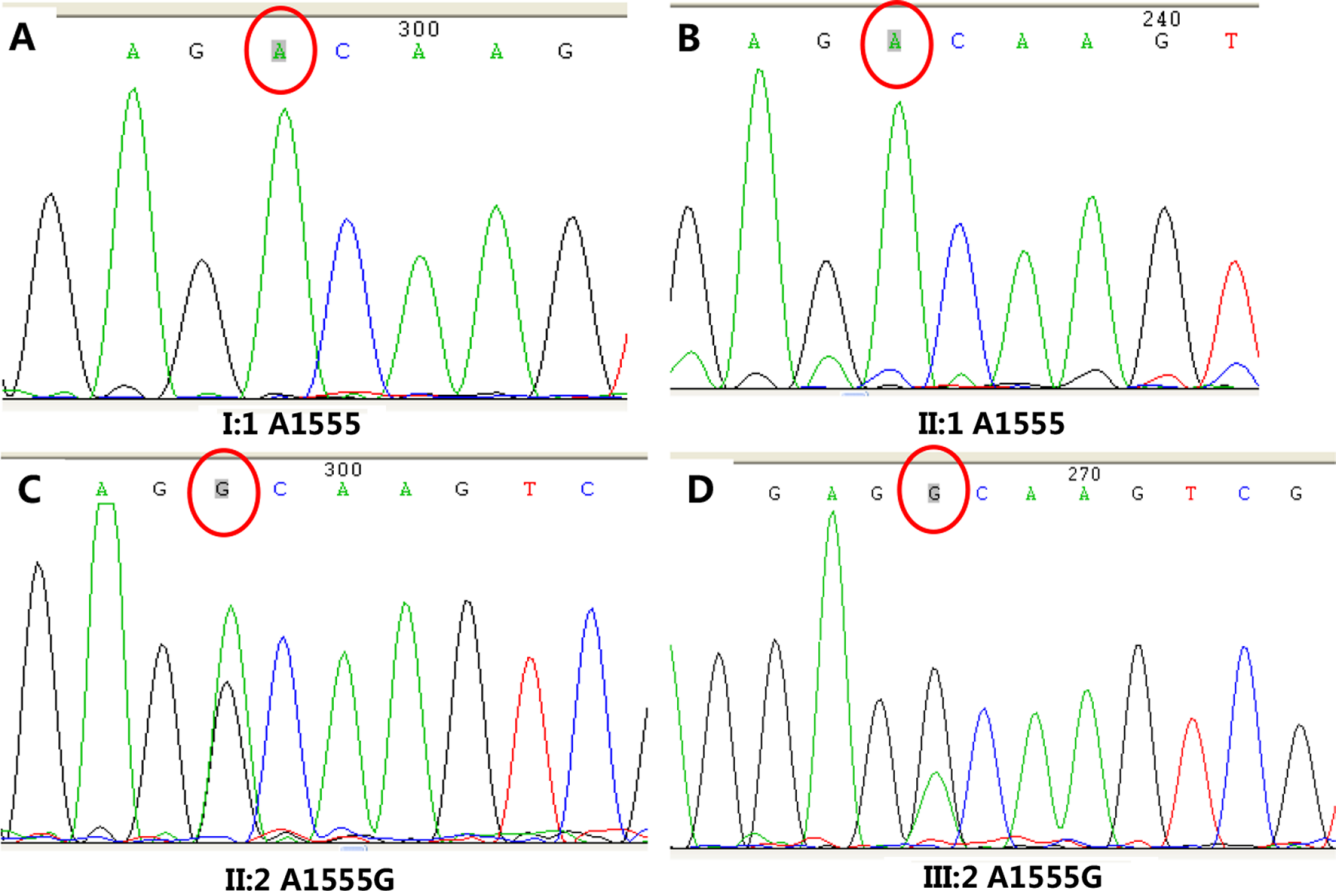
Results of sanger sequencing. **A** I:1 without A1555G. **B** II:1 without A1555G. **C** II:2 with A1555G heteroplasmic mutation. **D** III:2 with A1555G heteroplasmic mutation

Family 2362 is a three-generation Chinese family, in which one member has severe sensorineural hearing loss (Fig. 1). To clarify the cause of deafness in this family member, we searched for the gene responsible for the deafness in the proband and her parents. The proband (III:1) had a compound heterozygous mutation in *GJB2* (c.176del16 and c.299delAT); the mother (II:3) carried the c.176del16 mutation, the father (II:1) carried the c.299delAT mutation, and all three(II:1, II:3, III:1) lacked the A1555G mutation. To identify carriers of the c.299delAT mutation, family member II:2 with normal hearing was also tested for deafness genes. II:2 did not carry the c.299delAT mutation but had an A1555G heteroplasmic mutation in the mitochondrial gene, detected by Sanger sequencing. Further tests revealed that III:2 also carried the A1555G heteroplasmic mutation, whereas I:1 lacked this mutation (Figs. 1, 2). Therefore, this study focused on the maternal lineage I:1, II:2, II:3, and III:2 to clarify the status of the A1555G mutation.

### Paternity testing

The paternity of the three generations (I:1, II:1, II:2, and III:2) was confirmed by genotype analysis of 15 informative STRs of chromosomal DNA, which yielded probabilities of paternity of 0.999999, assuming a prior probability of 0.50. Therefore, the family members were all related.

### Quantitative analysis of the A1555G mutation

Targeted NGS showed that the subject (II:2) carried a heterogeneous A1555G mutation, at 28.68% heteroplasmy. The son of II:2 (III:2) was also a heterogeneous mutation carrier, with a level of heteroplasmy of 7.25%. The brother (II:1) and mother (I:1) of II:2 had 0.23% and 0.03% A1555G heteroplasmy, respectively (Table 1).

**Table1:** A1555G mutation of the maternal members in F2362

Subject	Gender	Age (years)	Results of Sanger sequence	Results of NGS (G/A) (%)
I:1	Female	60	(–)	0.03
II:1	Male	33	(–)	0.23
II:2	Female	31	(+)	28.68
III:2	Male	1	(+)	7.25

The level of mitochondrial 12S rRNA A1555G heteroplasmy in all 100 controls with normal hearing (Group 1) and in 100 patients with severe sensorineural hearing loss (Group 2) was less than 1% and did not differ significantly between these two control groups (*P* > 0.05; Table 2).

**Table2:** Summary of A1555G mutation for two control groups

Group	Number	Hearing	Heteroplasmy level (%)		
			Min	Max	Mean
1	100	Normal	0.03	0.26	0.11
2	100	Sensorinural hearing loss	0.01	0.31	0.10

## Discussion

The mitochondrial 12S rRNA A1555G mutation is a hot spot for deafness-associated mutations in the Chinese population [8]. This mutation has been detected in up to 60% of hearing-impaired patients with previous exposure to aminoglycosides and in 0.09–0.70% of the general population [9, 10]. The incidence of this mutation is much lower in nonsyndromic hearing loss than in aminoglycoside-induced hearing impairment. In a Chinese pediatric population, Lu et al. reported that the incidence of the A1555G mutation was 1.43% in nonsyndromic hearing loss and 10.41% in aminoglycoside-induced hearing loss [11]. Although the contribution of the mtDNA A1555G mutation to congenital (prelingual, early childhood onset) deafness is minor, mitochondrial involvement is seen in patients with postlingual hearing impairment, a much larger population.

There is no treatment for mitochondrial hearing impairment, suggesting that testing for the mutation should be performed routinely before administering aminoglycoside antibiotics. Detection of the A1555G mutation is an important part of deafness gene screening. Sanger sequencing was long the gold standard for identifying unknown mtDNA point mutations before the advent of massively parallel sequencing analysis. However, the conventional Sanger sequencing does not have the sensitivity to detect heteroplasmic mutations below about 20%. NGS technologies have the capability of massively parallel sequencing and offer a robust platform for comprehensive analysis of mtDNA [12]. The small size of the mitochondrial genome resulting in high coverage at each nucleotide position enable more rapid, sensitive, and accurate quantification of low-level heteroplasmy. NGS is a powerful tool for detecting low-level heteroplasmy variants in the mitochondrial genome, which has greatly improved the ability to distinguish carriers from non-carriers. The sensitive detection and accurate quantification of pathogenic heteroplasmic changes are helpful for risk prediction and genetic counseling.

In our study, targeted NGS showed that subject II:2 is a heterogeneous carrier of the mitochondrial 12S rRNA A1555G mutation at a level of 28.68% heteroplasmy. The son of II:2 (III:2) is also a heterogeneous mutation carrier at a level of 7.25% heteroplasmy. The brother (II:1) and mother (I:1) had 0.23% and 0.03% A1555G heteroplasmy. The level of mitochondrial 12S rRNA A1555G heteroplasmy tested by NGS in the 100 cases with normal hearing and 100 patients with severe sensorineural hearing loss negative for *GJB2*, *SLC26A4*, and mtDNA 12S rRNA was less than 1% and did not differ significantly (*P* > 0.05) between these two groups.

The presence of the mtDNA mutation in subject II:2 but not in her mother has three possible explanations: (1) other family members in the maternal lineage harbor the mutation, but at levels below the detection limit; (2) there is no biological relationship between subject II:2 and other family members tested; or (3) a de novo mutation occurred. We investigated all three possibilities.

To investigate the possibility that the mutation is present at a level below the sensitivity of the sequencing method, we sequenced the mtDNA at an average depth of 5000 × using NGS technology. The Ion Proton sequencing platform is sensitive enough to detect point mutations present with heteroplasmy levels as low as 1%. In the control groups, the level of mitochondrial 12S rRNA A1555G heteroplasmy was less than 1%, confirming the accuracy of this method. The probability of multiple false-negative results in the brother (II:1) and mother (I:1) is very low. Therefore, it is unlikely that other family members in the maternal lineage also carry the mutation at heteroplasmy levels below the detection limit.

To investigate non-kinship as the potential cause, we confirmed kinship from the mothers within the family. The paternity of the three generations (I:1, II:1, II:2, and III:2) was confirmed by genotype analysis of 15 informative STRs, yielding a probability of paternity of 0.999999, assuming a prior probability of 0.50. Having excluded kinship and sensitivity issues, we conclude that the A1555G mutation most likely appeared de novo in the family.

The A1555G mutation has been detected in patients with different haplotypes, indicating that de novo appearance of the A1555G mutation has occurred frequently in the past. MtDNA is solely inherited maternally and does not recombine, consequently, mutations accumulate in maternal lineages. According to most literature reports, mitochondria have relatively less sophisticated DNA protection and repair systems and high mutation rates [13]. But there are very few reports about de novo mutation in mitochondria, so the frequency of de novo mutation in mitochondria is not clear. In our previous study, we found that among 193 families carrying mitochondrial A1555G mutation, only one family had de novo mutation. So the frequency of de novo mutation in mitochondrial A1555G is 0.52% based on our data. Although the mechanism behind the de novo appearance of the A1555G mutation is unknown, we speculate that the mutation likely occurred during oogenesis (during embryonic development of the mother) or during early embryonic development of subject II:2.

There has been considerable debate about whether paternal mitochondrial DNA (mtDNA) transmission may coexist with maternal transmission of mtDNA, it is generally believed that mitochondria and mtDNA are exclusively maternally inherited in humans. This study did not verify the mitochondrial genetic maternal model. In the future research, we will supplement this part of work. In Fig. 1, we show the genetic relationship between I:1/II:2 and II:2/III:2 according to the law of maternal inheritance.

The observation of a de novo A1555G mutation is relevant for both diagnostic investigations and genetic counselling. First, even when there is no (maternal) family history in patients with deafness, A1555G mutation screening should still be performed to identify the cause of the disease. Second, screening in maternally related family members is recommended to provide reliable counselling for these families, given that the A1555G mutation may have arisen de novo. A genetic diagnosis of the A1555G mutation in an isolated patient does not necessarily mean that others in the maternal lineage also harbor the mutation. Although the vast majority of A1555G mutations are inherited maternally, a thorough family investigation should always be performed. Genetic counselling for deafness caused by the A1555G mutation is complicated. In individuals treated with aminoglycosides and thus at risk of hearing loss, mtDNA analysis can help predict hearing loss and the need to take precautions before symptom onset, as well as enable more accurate genetic counseling.

## Conclusion

We identified a family in whom the A1555G mutation appears to have arisen de novo. This has importance for both diagnosing and counselling patients. Determining if a mutation is inherited or de novo affects counseling regarding the risk of recurrence. This is the first report of a family with a de novo A1555G mutation, and it provides additional information on the origin and inheritance of the A1555G mutation.

## Data Availability

All data and material are available on the database of the Genetic Testing Center for Deafness (PLA General Hospital, Beijing, China).The data that support the findings of this study are openly available in https://dataview.ncbi.nlm.nih.gov, accession number: PRJNA830478.

## Abbreviations

mtDNA: Mitochondrial DNA
NGS: Next-generation sequencing
STRs: Short tandem repeats
PCR: Polymerase chain reaction
